# Three‐unit fixed dental prostheses supported by either two abutment implants or two abutment teeth: A comparative retrospective cohort study

**DOI:** 10.1002/cre2.562

**Published:** 2022-04-05

**Authors:** Christiaan W. P. Pol, Gerry M. Raghoebar, Marco S. Cune, Henny J. A. Meijer

**Affiliations:** ^1^ Department of Oral Surgery and Implant Dentistry, Center for Dentistry and Oral Hygiene, University of Groningen University Medical Center Groningen Groningen The Netherlands; ^2^ Department of Oral and Maxillofacial Surgery, University of Groningen University Medical Center Groningen Groningen The Netherlands; ^3^ Department of Oral and Maxillofacial Surgery, Prosthodontics and Special Dental Care St. Antonius Hospital Nieuwegein Nieuwegein The Netherlands; ^4^ Department of Oral and Maxillofacial Surgery, Prosthodontics and Special Dental Care, University of Utrecht University Medical Center Utrecht Utrecht The Netherlands

**Keywords:** cohort study, dental implants, fixed dental prostheses, fixed partial denture, prosthesis failure

## Abstract

**Objectives:**

In general, similar restorative constructions are made on natural teeth and on dental implants. The assumption is made that implants and their restoration perform the same as natural roots and their prosthetic restoration. Evaluating cohorts of three‐unit bridges on teeth and on implants, this retrospective clinical study aimed to compare implants and teeth as supporting units, including the reconstructions, in terms of survival, success, clinical, radiographic, and patient‐reported outcomes.

**Material and Methods:**

From an 8‐year period, all patients treated with a posterior three‐unit fixed reconstruction on either implants or teeth, with a follow‐up of at least 2 years, were identified. For each implant‐supported reconstruction, a comparable tooth‐supported reconstruction was selected, based on the length of follow‐up, the material of the reconstruction, and the location in either the maxilla or mandible.

**Results:**

For the Implant‐group, 24 patients could be matched with 24 best matching patients with tooth‐supported fixed dental prostheses (FPDs). Supporting implants and implant‐supported reconstructions were all in function with a mean follow‐up of 52 ± 23 months. Two tooth‐supported reconstructions had been replaced (91.7% survival) (mean follow‐up: 52 ± 19 months). Radiographic bone levels and soft tissue conditions were favorable in both groups with minor differences. There was no significant difference in overall patient satisfaction. The modified USPHS‐score revealed an 87.5% overall success in the Implant‐group and 91.7% in the Tooth‐group.

**Conclusions:**

Implant‐supported three‐unit FDPs are a reliable treatment option with survival and success rates not significantly different from the results of tooth‐supported three‐unit FDPs.

## 1 INTRODUCTION

Since the introduction of implants, many of the restorative materials and techniques that were first used successfully on natural teeth were applied to implants, under the assumption that they would elicit similar results. Although some materials and techniques are suggested to be more suitable to either treatment modality (Sailer et al., 2018), in most cases, the final reconstructions are still designed regardless of the nature of the supporting units. However, there are biological and technical differences between the implant‐ and tooth‐retained designs. Teeth have a periodontal ligament to absorb functional forces while implants lack such a feature. Reconstructions on abutment teeth are always placed using cement, whereas in most cases, with implants a screw‐type connection is used, either between abutments and implants or between reconstructions and the implants. Applying the experience gained in tooth‐supported reconstructions to implant‐supported reconstructions has resulted in highly successful treatments. This generally holds true in both single‐ (Jung et al., 2012; Sailer et al., 2015) and multiunit cases (Pjetursson et al., 2012, 2015) The design of multiunit reconstructions differs greatly, ranging from two units to full‐arch reconstructions and the number of variables in biological and technical aspects introduced by spanning the arch or the anterior regions makes the comparison between cases difficult (Pjetursson et al., 2015; Sailer et al., 2018).

In a systematic review on zirconia‐based fixed dental prostheses (FDPs) on implants and teeth, it was reported that 5‐year results on both types of abutments were good and very much alike, but that comparative studies were necessary (Le et al., 2015). Another systematic review analyzed survival rates and complication rates of the tooth‐ and implant‐supported FPDs and reported similar kinds of survival and complications, but did not compare similar types of restorations (Pjetursson et al., 2007). A more recent systematic review on three‐unit posterior reconstructions did not reveal any studies performing a direct comparison between teeth and implants as supporting units, but, by comparing the available data, supported the claim that implants perform similar to teeth, with similarly high treatment survival and success (Pol et al., 2018).

As studies with a direct comparison are scarce, insight could also be gained by analyzing systematic reviews on either tooth‐or implant‐supported systematic reviews. Systematic reviews on tooth‐supported FDPs with more than one abutment tooth have been published by Pjetursson and, for cantilevered FDPs, by Aglietta (Aglietta et al., 2009; Pjetursson et al., 2015). Both of these reviews showed good results, with 5‐year FDP survival percentages of over 94%. A systematic review on implant‐supported FDPs with more than one abutment implant has been published by Pjetursson and showed good 5‐year results, with FDP survival rates of over 96% and a 97% survival rate of the supporting implants (Pjetursson et al., 2012). However, this review noted a lack of studies with a long‐term follow‐up and did not examine the results of three‐unit restorations specifically on two abutments, nor performed an analysis on the impact of the length of the prostheses on the survival or complication rates (Pjetursson et al., 2012).

Presently, there is a lack of comparative studies and patient‐reported outcome measures (PROMs) are underreported. Therefore, this study aims to use a study design with matched groups and evaluate, in a retrospective comparative clinical study, the results of cohorts of three‐unit bridges on teeth and on implants in terms of survival, success, clinical, radiographic, and PROMs.

## 2 MATERIALS AND METHODS

### 2.1 Design of the study

For this comparative retrospective cohort study, all patients treated with a posterior three‐unit FPD supported by two dental implants or two natural teeth, between January 1, 2009 and January 1, 2017, were identified. Treatments were performed in a University clinic. Dental implants were placed by staff; reconstructions either by staff or by dental students under the supervision of experienced staff members.

Posterior three‐unit FPDs supported by two dental implants were seen as test group while posterior three‐unit FDPs supported by two natural teeth served as a matched control group.

Criteria for a patient to be included in the study were as follows:

– patients with a three‐unit FDP on either two implants or two natural teeth in the (pre)molar region;
– treated between January 1, 2009 and January 1, 2017;
– FDPs should have a central pontic;
– a follow‐up period of at least 2 years after prosthetic reconstruction.

Exclusion criteria were:

– treated <18 years of age;
– no radiograph available from the start of clinical service of the reconstruction.

To enable the comparative study, the test group with posterior implant‐supported FDPs was matched with an equal number of patients of the control group with tooth‐supported FDPs. The selection was done manually by matching pairs of patients, based on the length of the follow‐up period, the material of the FPD, and the location (either maxilla or mandible) of the reconstruction, in that order; moreover, information regarding details on the patient or the course of the treatment, including success or survival of the supporting units or the reconstruction, other than the factors used in matching the cases, was kept confidential.

This study, for which data collection took part at regular routine control visits and without collection of extra data or additional burden to the patient, did not require clearance from the Medical Ethical Committee in accordance with the Medical Research Involving Human Subjects Act (WMO). This study was conducted in accordance with the requirements of the Helsinki Declaration of 1975 and revised in 2008 and CONSORT Guidelines. Written informed consent was obtained from all eligible patients before enrollment.

The patient files of all patients selected were screened for information regarding the reconstruction. Any complications reported during the follow‐up period were listed. Failure of a supporting implant or tooth was defined as the removal of an implant or tooth due to any technical or biological complications. A reconstruction was considered a failure if it was removed permanently from the patient or was damaged beyond repair following technical complications. If a failure had occurred during the follow‐up period, the moment of failure was noted. All patients with the reconstruction still in function, or with unknown status of the reconstruction, were informed about the study and examined during the yearly regular visit. During the clinical examination, the reconstruction, the supporting units, and the surrounding hard and soft tissues were evaluated and patient satisfaction with the reconstruction was assessed.

### 2.2 Outcome measures

The following outcome measures were assessed:

– survival of the implants/teeth;
– survival of the reconstruction;
– condition of the hard and soft tissues surrounding the supporting units;
– patient‐reported outcome measures (PROMs);
– the quality of the reconstruction using modified United States Public Health Service (USPHS) criteria.

The clinical examination of the condition of the soft tissues surrounding the supporting units was performed using the following criteria:

– assessment of plaque accumulation with the modified Plaque Index (Mombelli et al., 1987);
– assessment of bleeding tendency with the modified Sulcus Index (Mombelli et al., 1987);
– assessment of peri‐implant inflammation with the Gingival Index (Loe & Silness, 1963);
– probing pocket depth: measured to the nearest millimeter using a manual periodontal probe.

Digital intra‐oral radiographs were taken using a parallel technique to evaluate the bone levels at the supporting units and the marginal adaptation of the reconstruction. The peri‐apical or peri‐implant region was inspected for bone lesions. Bone levels at follow‐up were compared to the radiograph taken after placement of the restoration. For each support, the bone loss was registered as the worst value, both at the distal and mesial support of the three‐unit FDP, of the change between the radiographs. A prosthodontist, familiar with the software measurement programs, performed the linear measurements on the digital radiographs.

In implant‐supported cases, using specially designed computer software (DicomWorks, Department of Biomedical Engineering, University Medical Center Groningen, The Netherlands), calibration was carried out in the horizontal and vertical plane for each radiograph by using the known dimensions of the implants to ensure correct measurement. Peri‐implant bone level changes were determined by measuring, both mesially and distally, the distance from the implant reference point (the junction between the implant and the abutment) to the level of the margin of the crestal bone (Pol et al., 2020; Sewerin, 1990).

In tooth‐supported cases, measurements were performed using the measurement function in the Novadent NovaX software package (version 7.14.2.6750; Complan Valens b.v., Hoorn, The Netherlands). The known dimensions of the radiographic sensor were used to convert measurements from pixels to millimeters. Calibration between the two time points was established by measuring the thickness of the major connectors, both mesial and distal of the central pontic, at the narrowest point. Then, on the mesial and distal sides of both supporting teeth, the distance between the radiographic bone level and the lowest projected edge of the FDP was measured (de Faria Vasconcelos et al., 2012; Misch et al., 2006; Vandenberghe et al., 2008).

The quality of the reconstruction was assessed according to the modified version of the USPHS criteria (Naenni et al., 2015; Pol et al., 2020; Spies et al., 2018) In these criteria, framework fracture, veneering fracture, loosening of the restoration (cement and/or screw), loss of the screw access hole restoration (in implant cases), occlusal wear, clinical marginal adaptation, anatomical form, restoration color, radiographic marginal adaptation, and patient satisfaction were evaluated as items defining the quality of the restoration, as indicated by a score ranging from “Alpha” to “Delta.”

A reconstruction was considered to be successful if all aspects scored only in the “Alpha” or “Bravo” categories. Reconstruction with one or more “Charlie” scores was considered unsuccessful but surviving. Reconstructions scoring “Delta” on one or more items were considered a failure and thus also unsuccessful. Any fracturing, loosening, or loss of screw access reconstruction reported during the observation period, was also included in the evaluation.

Patients' satisfaction was assessed with the questionnaire used by (Telleman et al., 2013). The patients were asked to respond to a series of statements regarding their dental situation, feelings, esthetics, and function, with answers on a five‐point rating scale ranging from “very dissatisfied” or “not in agreement” (score 1) to “very satisfied” or “in agreement” (score 5). Furthermore, patients were asked to rate overall satisfaction concerning their dental situation on a scale from 1 (lowest satisfaction) to 10 (highest satisfaction). Patients were asked to complete the questionnaire in a separate room, without supervision. The questionnaire was collected by an assistant not involved in the research appointment of the patient.

For use in the modified USPHS evaluation, the overall patient satisfaction rating was converted. Inhabitants of the Netherlands are used to ratings of 1–10, as the same rating methodology is used in education ratings. In this rating lower than 6 is not sufficient, 6 means sufficient but definitely not a success, 7 means sufficient and promising, and the rating 8 means definitely a success. For meaningful conversion to the modified USPHS evaluation, the rating of 5 or lower was considered as failure (“Delta”), 6 as unsuccessful (“Charlie”), 7 as moderately successful (“Bravo”), and 8 or higher as successful (“Alpha”).

### 2.3 Statistical method

The analyses were performed with IBM SPSS Statistics 23.0 (IBM Corporation, Chicago, IL, USA). A significance level of .05 was chosen for all the tests.

Variables presenting with a normal data distribution as identified by the Shapiro–Wilk test, were analyzed by independent‐samples *t* test. Between group comparisons were performed using the Mann–Whitney *U* test.

## 3 RESULTS

Totally, 30 patients were identified who were treated with a posterior three‐unit FPD supported by two dental implants and meeting the inclusion criteria. From this group, six patients were unable to attend the follow‐up visit: two patients died, two were unable to attend due to health‐related issues and two patients moved without leaving a new address.

**Figure 1:**
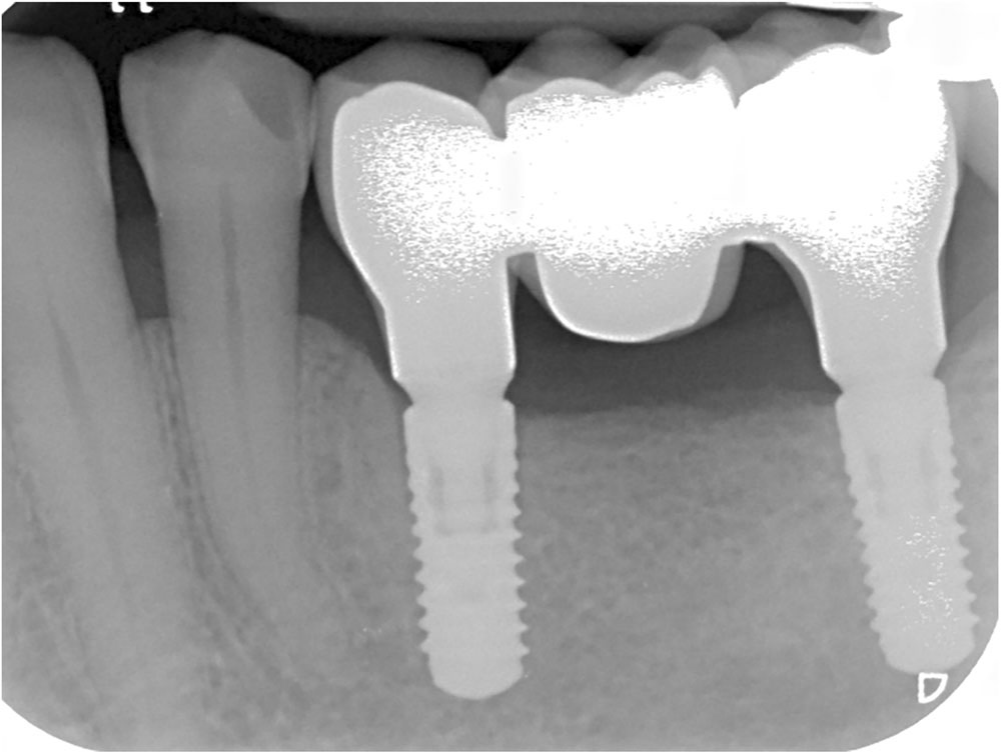
Sample radiograph of implant‐supported fixed dental prostheses, after a follow‐period of 2 years

**Figure 2:**
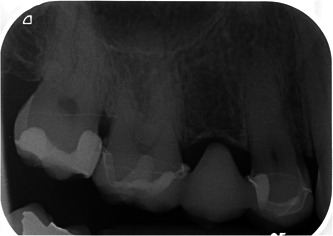
Sample radiograph of tooth‐supported fixed dental prostheses, after a follow‐period of 4 years

The remaining 24 cases (Implant‐group, Figure 1) were paired with the 24 best matching patients with a tooth‐supported posterior three‐unit FPD (Tooth‐group, Figure 2). These best matching cases were selected from the compiled list of all treated patients from the same time period (*n* = 167) based on the length of follow‐up, the material of the reconstruction, and the position in the maxilla or mandible. All implants were from the Straumann dental implant system; all implants having a sandblasted, large‐grit, acid‐etched surface, bone‐level, and tissue‐level type implants were equally distributed and all implant restorations were screw‐retained.

The characteristics of the patients included in both study groups are depicted in Table 1. Only the patient's age at the time of treatment differed significantly between the groups (*p* < .001) with the Implant‐group having the higher age at treatment.

**Table 1:** Characteristics of the patients in the study groups

Patients	Implants (test)	Teeth (control)	*p* Value
*n*	24	24	
Male	7 (29.2%)	13 (54.2%)	.08
Female	17 (70.8%)	11 (45.8%)	
Follow‐up (months) Mean ± SD [range]	52 ± 23 [24–89]	52 ± 19 [27–76]	1.00
FDP material^a^			1.00
PFM	12	11	
PFZ	11	12	
FZ	1	1	
Mandible	9	11	.77
Maxilla	15	13	
Patient age at treatment (years)			*<.001*
Average ± SD [range]	71 ± 7 [63–86]	58 ± 13 [26–76]	

^a^ Fixed dental prosthesis (FDP) consisted of porcelain fused to metal (PFM), porcelain fused to zirconia (PFZ), or full zirconia (FZ).

In both implant‐ and tooth‐supported cases, all supporting units of the selected patients were in function at the follow‐up visit (100% survival of supporting units). All 24 implant‐supported FDPs survived until the follow‐up visit (100% survival), whereas in the tooth‐supported group, 22 of the 24 FDPs were in function (91.7% survival). Two restorations in the Tooth‐group had been replaced before the follow‐up examination and were thus scored as failures: one FDP was replaced after 5 months in service because of impaired marginal adaptation resulting in sensitivity in one of the supporting teeth; another FDP was replaced after 12 months, also due to impaired marginal adaptation. Both reconstructions were successfully replaced and, in both cases, the supporting teeth and new reconstruction continued to function without problems.

The condition of the soft tissues surrounding the supports was favorable in both groups (Table 2). There were no significant differences between the groups, except for plaque (more plaque at the distal support in the Tooth‐group: *p* = .031) and bleeding (more bleeding at the mesial support in the Implant‐group: *p* = .017). In tooth‐supported cases, no signs of inflammation (e.g., periapical radiopacity) or caries were found on the X‐ray images of the supporting units.

**Table 2:** Comparison of clinical and radiographical variable scores and frequency distribution between the study groups

Variable	Implant (test), *n* = 24	Teeth (control), *n* = 22	*p* Value
Plaque index			
Mesial support	0 = 21	0 = 15	.219
	1 = 3	1 = 5	
	2 = 0	2 = 2	
Distal support	0 = 21	0 = 12	*.031*
	1 = 3	1 = 6	
	2 = 0	2 = 4	
Bleeding index			
Mesial support	0 = 10	0 = 17	*.017*
	1 = 8	1 = 2	
	2 = 6	2 = 3	
	3 = 2	3 = 1	
Distal support	0 = 9	0 = 14	.638
	1 = 8	1 = 5	
	2 = 6	2 = 4	
	3 = 2	3 = 1	
Gingiva index			
Mesial support	0 = 22	0 = 18	.581
	1 = 2	1 = 4	
Distal support	0 = 22	0 = 18	.581
	1 = 2	1 = 4	
Probing pocket depth			
Mesial support (in mm)	2 = 1		.609
	3 = 18	3 = 15	
	4 = 4	4 = 5	
	5 = 1		
Distal support (in mm)	2 = 1	2 = 1	.578
	3 = 14	3 = 10	
	4 = 6	4 = 6	
	5 = 3	5 = 2	
Radiographical bone level			
Mesial support (in mm)	−0.70 ± 0.70	−0.33 ± 0.44	*.033*
Distal support (in mm)	−0.51 ± 0.48	−0.31 ± 0.44	.164

The mean marginal bone level change in the Implant‐group was −0.70 ± 0.70 mm at the mesial support and −0.51 ± 0.48 at the distal support. The mean marginal bone level change in the Tooth‐group was −0.33 ± 0.44 mm at the mesial support and −0.31 ± 0.44 at the distal support. There was no significant difference between groups at the distal support (*p* = .164), at the mesial support bone loss was significantly larger in implant cases (*p* = .033). Within both groups, the difference in the bone loss was not significant between mesial and distal support (*p* = .904 for Implant‐group and *p* = .274 for Tooth‐group).

There was no significant difference in overall patient satisfaction between both groups (*p* = .64) (Table 3). The modified USPHS‐score, revealed an overall success, scoring either Alpha or Bravo, in the Implant‐group of 87.5% and in the Tooth‐group of 91.7% (Table 4).

**Table 3:** Comparison of patient‐reported outcomes and satisfaction scores between groups

	% in agreement		
	Implant (test), *n* = 24	Teeth (control), *n* = 22	*p* Value
Feelings			
Feeling ashamed	4.2%	0.0%	.20
Self‐confidence increased	29.2%	31.2%	.82
Visible having an FPD	0.0%	0.0%	.75
Function			
Evade eating with the restoration	12.5%	0.0%	.80
The ability to chew did not improve	8.3%	27.3%	.22
Aesthetics			
Not satisfied with the color of the restoration	4.2%	0.0%	.29
Not satisfied with the shape of the restoration	8.3%	0.0%	.72
Overall satisfaction (0–10) ± SD	9.0 ± 1.1	8.5 ± 1.0	.64

**Table 4:** Modified USPHS criteria for evaluation of the restoration during the follow‐up period

USPHS criteria (Implant, *n* = 24/Teeth, *n* = 24)^a^	Alpha (A)	Bravo (B)	Charlie (C)	Delta (D)
Framework fracture	No fracture of framework	‐	‐	Fracture of framework
	Implant: 24 (100%)			Implant: 0 (0%)
	Teeth: 24 (100%)			Teeth: 0 (0%)
Veneering fracture	No fracture	Chipping but polishing possible	Chipping down to framework (repair needed)	New reconstruction is mandatory
	Implant: 18 (75%)	Implant: 6 (25%)	Implant: 0 (0%)	Implant: 0 (0%)
	Teeth: 22 (91.7%)	Teeth: 2 (8.3%)	Teeth: 0 (0%)	Teeth: 0 (0%)
Loosening of the restoration (cement and/or screw)	No loosening	‐	Repositioning possible	Repositioning not possible—new reconstruction is needed
	Implant: 23 (95.8%)		Implant: 1 (4.2%)	Implant: (0%)
	Teeth: 24 (100%)		Teeth: (0%)	Teeth: (0%)
Screw access hole restoration	No loss of restoration	‐	Restoration lost (repairable)	‐
	Implant: 24 (100%)		Implant: 0 (0%)	
	Teeth: n/a		Teeth: n/a	
Occlusal wear^a^	No wear facets on restoration and opposing teeth	Small wear facets (diameter < 2 mm) on restoration and/or opposing teeth	Wear facets (diameter > 2 mm) on restoration and/or opposing teeth	New reconstruction is needed
	Implant: 23 (95.8%)	Implant: 1 (4.2%)	Implant: 0 (0%)	Implant: 0 (0%)
	Teeth: 21 (95.5%)	Teeth: 1 (4.5%)	Teeth: 0 (0%)	Teeth: 0 (0%)
Marginal adaptation	Probe does not catch	Probe catches slightly, but no gap detectable	Gap with cement exposure	New reconstruction is needed
	Implant: 24 (100%)	Implant: 0 (0%)	Implant: 0 (0%)	Implant: 0 (0%)
	Teeth: 21 (87.5%)	Teeth: 1 (4.2%)	Teeth: 0 (0%)	Teeth: 2 (8.3%)
Anatomical form^a^	Ideal anatomical shape, good proximal contacts	Slightly over or under contoured, weak proximal contacts	Highly over or under contoured, open proximal contacts	New reconstruction is needed
	Implant: 20 (83.3%)	Implant: 3 (12.5%)	Implant: 1 (4.2%)	Implant: 0 (0%)
	Teeth: 22 (100%)	Teeth: 0 (0%)	Teeth: 0 (0%)	Teeth: 0 (0%)
Radiographs^a^	No visible cementation gap on X‐ray	Minor gap visible	Major gap visible—new reconstruction not needed	Major gap visible—New reconstruction needed
	Implant: 23 (95.8%)	Implant: 1 (4.2%)	Implant: (0%)	Implant: (0%)
	Teeth: 21 (95.5%)	Teeth: 1 (4.5%)	Teeth: 0 (0%)	Teeth: 0 (0%)
Patient satisfaction^a^	Very satisfied	Moderately satisfied	Not satisfied—new reconstruction not needed	Not satisfied—new reconstruction needed
	Implant: 21 (91.3%)	Implant: 2 (8.3%)	Implant: 1 (4.2%)	Implant: 0 (0%)
	Teeth: 19 (86.4%)	Teeth: 3 (13.6%)	Teeth: 0 (0%)	Teeth: 0 (0%)
Overall (worst value per FDP)	Success and survival	Success and survival (impaired)	Survival	Failure
	Implant: 11 (45.8%)	Implant: 10 (41.7%)	Implant: 3 (12.5%)	Implant: 0 (0%)
	Teeth: 16 (66.7%)	Teeth: 6 (25.0%)	Teeth: 0 (0%)	Teeth: 2 (8.3%)

^a^ These items were examined at follow‐up visit and are thus presented for the 22 remaining teeth‐supported FDPs, other items at follow‐up or until failure occurred.

## 4 DISCUSSION

To the best of our knowledge, this is the first study comparing three‐unit fixed dental reconstructions supported by either two implants or two teeth. Both study groups show more or less similar results, suggesting that FDPs either on two implants or two teeth act alike.

No prior studies with a direct comparison between teeth and implants in this similar situation were identified by recent systematic reviews (Le et al., 2015; Pjetursson et al., 2007; Pol et al., 2018). By combining the available evidence Pol et al. drew the conclusion that teeth and implants perform very similarly when supporting three‐unit posterior reconstructions (Pol et al., 2018). These systematic reviews point out that there is a limited amount of evidence available with longer follow‐up on larger groups of patients and that data on clinical aspects and patient‐reported outcome measures is scarce, whereas studies with a direct comparison of teeth to implants in this particular indication are nonexistent (Le et al., 2015; Pjetursson et al., 2007; Pol et al., 2018).

It must be mentioned that the range in the follow‐up period in the present study is rather large (Table 1). However, due to the fact that this is the case in both groups and not significantly different, it can be assumed that it does not affect the comparison.

The literature reports an approximated annual survival rate of the supporting teeth or implants of 99% versus 98.7% over a mean follow‐up period of 41 months (Pol et al., 2018). In the present study, all supporting units survived (100% survival of both teeth and implants) after a follow‐up of up to 89 months (average 52 months).

From the literature, an FDP survival rate of 96.4% and 97.4% per year is expected for tooth and implant reconstructions (Pol et al., 2018). In the present study 22 of the 24 tooth‐supported FDPs were in function after an average of 52 months (91.7% survival after follow‐up or 98.1% per year of follow‐up) and all 24 implant‐supported FDPs survived until the follow‐up visit (100% survival). Thus, results from the present study are above average for both survival of supporting units and FDP.

The literature provides only limited information on soft tissue conditions, as indicated by the systematic reviews, with low levels of reporting of variables (Le et al., 2015; Pjetursson et al., 2007; Pol et al., 2018). In this study, the condition of soft tissues surrounding the supports was found to be favorable, with only a limited number of significant differences between the groups. There was slightly more plaque on the distal supports in the Tooth‐group and more bleeding at the mesial support in the Implant‐group. This might be indicative of some regions of reconstruction being more difficult to clean: the distal support in tooth‐supported FDPs and the interproximal region for implant‐supported reconstructions.

In implant publications, the peri‐implant bone level change is a commonly reported variable (Le et al., 2015; Pjetursson et al., 2007; Pol et al., 2018). For tooth‐supported reconstructions, the bone level change is rarely reported (Pol et al., 2018). This study aimed to address this shortcoming by building on methods suggested in the literature to measure radiographs in tooth‐supported cases (de Faria Vasconcelos et al., 2012; Misch et al., 2006; Vandenberghe et al., 2008). For teeth, measurement is hindered, as there is no easy way to calibrate between radiographs without an easily identifiable object of known and stable dimensions, while in implant cases, radiographic measurements can be calibrated by using the stable implant dimensions (Pol et al., 2020; Sewerin, 1990).

Neither the difference in the bone loss within both groups between the mesial and the distal support, nor the difference between both groups for the distal supper was significant, but a significant difference was found between the bone level change in both groups at the mesial support. A recent systematic review, specifically on posterior three‐unit FDPs, revealed no publications on bone loss in tooth‐supported cases for comparison of the outcome, but results in the Implant‐group appear to be in line with other publications (Pol et al., 2018, 2020). However, the clinical significance is limited, as the bone loss in both groups is too small to cause the loss of a supporting unit.

An important evaluation of interventions should be patient satisfaction, as after spending time and money to solve a dental handicap, serious enough to seek treatment, the patient should be satisfied with the result of this treatment. Nonetheless, it was found that a high percentage of studies do not report, in any way, patient satisfaction. In the present study, PROMs were assessed with a questionnaire used by Telleman et al. (2013), together with an overall satisfaction score from 1 to 10. The choice for this questionnaire was based on the fact that it is explicitly suitable for the posterior region because it addresses not only esthetics but also the ability to chew, comfort, and pain during eating. The overall satisfaction score from 1 to 10 is very clear in the Netherlands as the same scoring methodology is used in education ratings. In this study, patients proved to be highly satisfied with treatment—with all but one patient reporting to be satisfied. The difference between groups was not significant. Patient satisfaction levels did not prompt any intervention in this study, while insufficient data was available in systematic reviews to compare this outcome to the literature (De Bruyn et al., 2015; Pol et al., 2018).

To assess the treatment results, a system based on the USPHS method was used. The original screening form is expanded over the years by several authors (Naenni et al., 2015; Spies et al., 2018). The version used was previously published and encompasses the most important outcomes of prosthetic treatment, including many common complications during follow‐up (Pol et al., 2020). By using this form to score a treatment, either when placing the reconstruction or at follow‐up visits, comparing prosthetic outcomes of various treatments becomes feasible. While some researchers have already used this or a very similar version, it could prove beneficial in comparing prosthetic outcomes if more publications would use a standardized scoring system. The modified UPSHS evaluation in this study resulted in a very high score on success and survival. Some reconstructions (10 in the Implant‐group and 6 in the Tooth‐group) required some intervention during follow‐up, mainly polishing due to chipping of small wear facets. In the Implant‐group, one restoration scored a Charlie‐rating on the contour and proximal contacts, with three more scoring as Bravo—indicating that the anatomical form in implant restorations is more difficult to achieve, possibly due to the difference in emergence profile. In the Tooth‐group, two restorations were replaced due to impaired marginal adaptation. The level of repairable complications in both groups, especially the veneering fractures, was above the literature reported annual average of 2.42% for tooth‐supported and 1.94% for implant‐supported restorations, which after 52 months would indicate a rate of repairable complications of 10.5% and 8.4%, respectively. Since veneering fracture makes up the largest part of treatable complications, monolithic restoration materials, especially zirconia, could drastically reduce the number of prosthetic complications.

One of the limitations of this study is the limited number of cases in both groups. As such, 24 patients should be enough in a group, but further subdivision into subgroups of different materials systems could disturb the comparison. However, it appeared that complications were limited in both groups. This justifies that conclusions could be based on the possible difference between “implant‐supported” and “tooth‐supported” and that different materials of the reconstructions have limited influence.

Another limitation of this study is the retrospective study design. The reconstructions of both groups were placed by various operators and in different circumstances, as a result of which there is no standardized treatment protocol. However, all reconstructions were made in the same university clinic by dental students with similar previous education and under the supervision of dentists experienced in both restorative dentistry and in supervising students. The results are indicative of the success of this supervision, as in both groups both the survival and success of the reconstructions are above average when compared to the literature.

Since reconstructions were done by dental students, some form of bias might arise in patient‐reported outcomes. Many patients sympathize with the students and this loyalty might prompt them to rate the reconstructions a little higher, although all reconstructions also perform well on more objectively measurable criteria. Both failures occurring in the Tooth‐group could possibly be attributed to the university environment: in both cases, the marginal adaptation was cause for replacement after a few months in service, although from the case files it was apparent in both cases that the marginal adaptation was judged to be impaired during initial placement of the restorations. Had these restorations been replaced before cementation, as would have been logical under clinical circumstances, these treatments would have been regarded as successful. Cementing these restorations was perhaps fueled by the desire of both student and staff to provide the patient with reconstruction after lengthy treatment, while also playing into the numerical requirements for students to complete treatments before graduating. Therefore, implementing a form of quality screening, such as (modified) USPHS‐criteria, in graduating requirements might be beneficial.

One limitation embedded in the very notion of this study design is the difference between the two patient groups. Patients missing three (or more) units in one region of the mouth received a three‐unit implant‐supported restoration, while patients in the Tooth‐group received treatment to replace one missing unit. Although receiving a larger reconstruction might possibly have a larger effect on the ability to chew, perceived oral handicap, or other factors impacting patient satisfaction, this was not supported by the data: no significant difference was found between groups on the patient satisfaction indicators. The difference between groups in the number of missing units was, most likely, the reason behind the only significant difference between study groups: the age of patients at treatment was significantly lower in the Tooth‐group, by 13 years. The tooth‐supported three‐unit restoration is an earlier step in the restorative cycle: based on the 96.4% annual failure rate reported in the literature, patients having a tooth‐supported reconstruction for 13 years have a 38% chance of this restoration failing during this period. If the supporting teeth would subsequently fail, the next restoration could include an implant‐supported restoration. The difference is also visible in the range of the age at treatment: not only was the average patient treated with implant‐based reconstruction older but the Implant‐group showed a smaller deviation, with a lower margin above the average age for tooth‐supported reconstructions.

## 5 CONCLUSION

Taking into account the limited number of participants in the groups and further subdivision into different materials, it could be concluded that implant‐supported three‐unit FDPs seem to be a reliable treatment with similar results as tooth‐supported three‐unit FDPs.

## AUTHOR CONTRIBUTIONS

Christiaan W. P. Pol and Henny J. A. Meijer designed the study, performed and supervised the execution, and wrote the manuscript in consultation with Gerry M. Raghoebar and Marco S. Cune.

## CONFLICTS OF INTEREST

The authors declare no conflicts of interest.

## Data Availability

The data that support the findings of this study are available from the corresponding author, C.P., upon reasonable request.
